# High-Frequency Rheological and Piezo-Voltage Waveform Characterization of Inkjet-Printed Polymer-Based Dopant-Source Inks

**DOI:** 10.3390/mi14010080

**Published:** 2022-12-28

**Authors:** Zulkifl Hussain, Zohreh Kiaee, Milad Nazarzadeh, Christian Reichel, Sebastian Tepner, Tri Tuladhar, Mike Jahn, Roman Keding

**Affiliations:** 1 Fraunhofer Institute for Solar Energy Systems ISE, Heidenhofstraße 2, 79110 Freiburg, Germany; 2 Trijet Limited, 59 Eland Way, Cambridge CB1 9XQ, UK

**Keywords:** inkjet printing, high-frequency rheology, polymer-based inks, phosphorus inks, boron inks

## Abstract

This work focuses on developing an understanding of the rheological properties of polymer-based dopant-source inks at the timescales relevant to inkjet printing and their corresponding roles in determining the production of defect-free droplets. Ink-specific optimization of printing processes for phosphorus and boron dopant-source inks with different compositions is demonstrated. Rheological flow curves measured by a piezo axial vibrator (PAV) were used to study the changes in complex viscosity (η*) and in the elastic (G′) and viscous (G″) components of the shear modulus (G*) with respect to changes in frequency (from f_min_ = 1 kHz to f_max_ = 10 kHz) to obtain an insight into the high-frequency behaviour of inks, as well as the effects of temperature (25 °C and 45 °C) and the natural aging time of the inks. Inks demonstrating complex viscosity η*_min_ ≥ 2 mPas to η*_max_ ≤ 20 mPas and an elastic modulus G′ ≤ 20 Pa, produced droplets with negligible defects. Of the three rheological parameters (η*, G′ and G″), the elastic component (G′) of the shear modulus was observed to have the greatest significance in determining the stability and homogeneity of ink droplets, thus dictating the quality of the printed structures. The reliability and stability of droplet formation were further investigated through voltage waveform simulation using an oscilloscope.

## 1. Introduction

Inkjet printing technology is very widely gaining acceptance at both the industrial and laboratory scale. Due to their economic viability and technical feasibility, printed electronics are expected to dominate the industry within a few decades, as indicated by the market share of printed, flexible, and organic electronics, which is estimated to grow from 41.2 billion USD in 2020 to 74 billion USD in 2030 [1]. Inkjet printing technology is enticing, primarily due to its flexibility, precision, digital printing templates, reduction in feature size down to 10–15 μm (using drop volumes as low as 1 PL) and non-contact mode of operation [2]. These advantages, especially the non-contact mode, have enabled the successful development of contamination-free, complex—yet precise—architectures, such as different functional layers in solar cells [3,4], thin-film transistors (TFTs) [5,6], sensors [7], etc. At the same time, inkjet printing can also be used for patterning or as an etch-dispensing process, such as in the etching of isolation dielectrics in the multi-step standard CMOS process [8]. Inkjet printing also has the flexibility to print droplets (as small as 33 µm in diameter, although smaller sizes can be achieved depending on the printhead), lines and full areas with very high precision [9]. The non-contact mode used by inkjet printing ensures minimal damage while guaranteeing the lowest contamination of printed structural elements and the lowest number of substrate defects and breakages. This is extremely important for applications such as high-efficiency solar cells utilizing thin substrates. The success of inkjet printing is largely determined by the viscoelastic properties of the inks, which can be measured through rheometry. The rheometry of complex fluids, such as polymer solutions or suspensions, can be studied judiciously using a piezo axial vibrator (PAV) rheometer, as explained by Crassous et al. [10]. Rheometry is indispensable, especially at the industrial level, given the paradigm shift in our understanding of the behaviour of polymer solutions (inks) at high frequency, which has been elegantly explained by Vadillo et al. [11] (for commercial inkjet fluids using PAV and torsional rheometers). Similar work has been undertaken in detail by Hoath et al. [12], where inkjet performance in drop-on-demand (DoD) printers (as used in this work) was linked to high-frequency rheological properties. For similar reasons, frequency ranges up to 10 kHz were used in this work. Moreover, in addition to overcoming the inertia effect in the device, which is commonly found at low frequency (approximately 100 Hz), high-frequency analysis reveals more local-scale dynamics compared to the collective large-scale phenomena reflected by low-frequency moduli. Since the operating conditions at the nozzle are dynamic, high-frequency (sweep or high shear rate) oscillatory measurements provide a better understanding of the real operating conditions [13].

In this study, phosphorus (P) and boron (B) dopant-source inks were used, which find applications in high-efficiency silicon solar cells [14], thin-film transistors [15], etc. Promising results for local inkjet printing with dopant-source inks incorporating tunnel oxide passivating contact (TOPCon) technology for Si solar cells have been reported [16,17]. Successful local passivating contacts on SiOx/Poly-Si have also been developed using P and B dopant-source inks [18]. However, in addition to the promising advantages offered by inkjet printing, there are some limitations that need to be addressed, such as cartridge-ink compatibility, nozzle clogging, droplet defects (coffee stain effects, threads, satellites), de-wetting, etc. Many of the mentioned defects are related to the rheology of inks. Therefore, to use these inks efficiently, it is imperative to understand the rheological behaviour of inks in the following terms:

- How do inks behave under mechanical stress?
- What are their responses to extended exposure to stress/increasing stress?
- How do these characteristics respond to changing temperature?
- What impact does aging have on the rheological properties?
- How much do they digress from the established laws of polymers?

## 2. Viscoelastic Properties and Printing Behaviour

**Figure 1:**
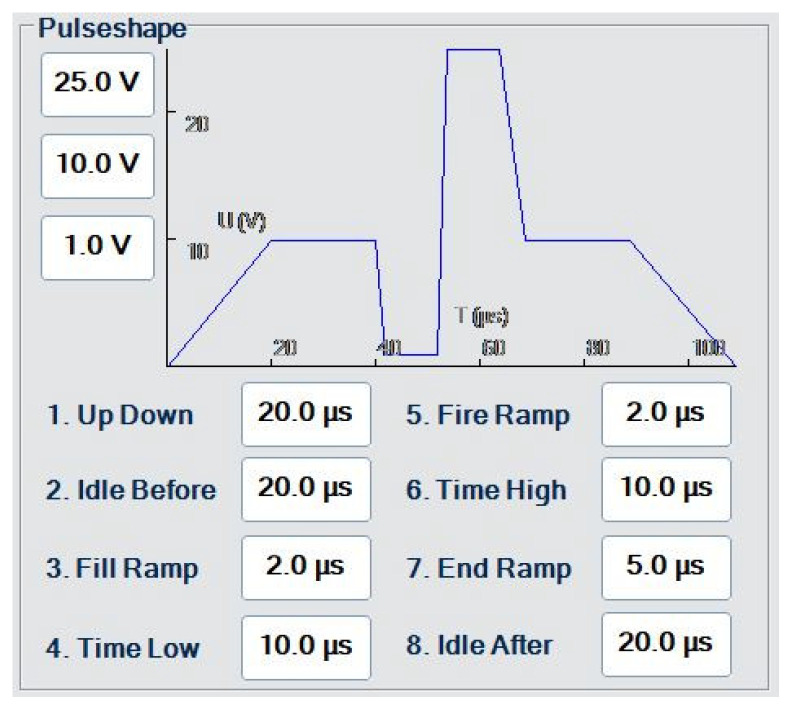
Default waveform of the PiXDRO LP50 inkjet printer from SUSS MicroTec [26], as used during printing.

The intrinsic properties relevant to the rheological behaviour of inks are the complex viscosity (η*) and the elastic (*G*′) and viscous (*G*″) components of the complex shear modulus (G*). These can be tuned, to some extent, according to inkjet-printing requirements through various external printhead parameters, such as the temperature, pulsed voltage waveform and firing frequency. The role of rheology appears as soon as the ink starts flowing through the printhead nozzles. For typical DoD inkjet printers, the diameter of the nozzles is in the range of 20–100 μm [19], and the firing frequency can be as high as 10 kHz [20] (jetting frequency at the piezo goes up to and higher than 30 kHz, depending on the time taken (approximately 24 µs) for steps 3 to 6 in Figure 1) or even much higher (depending on the droplet size and the application), which together make the operating conditions very conducive for high-shear-rate production. Furthermore, the firing frequency can exceed over 100 kHz in more advanced industrial printers, e.g., Dimatix Samba printheads [21]. Therefore, to understand the exact flow behaviour of inks, high-shear-rate rheological analysis becomes important. A PAV rheometer offers the possibility of studying the viscoelastic behaviour of inks by analysing them at higher frequencies (up to 10 kHz). This takes us a step closer in anticipating the printing behaviour. Complex viscosity is observed to play an indirect role in determining the overall efficiency of the process through the actuation voltage; the higher the viscosity of the ink, the higher its actuation voltage will be [11]. However, a higher voltage also implies a large droplet volume, which usually leads to reduced sharpness around the edges of the printed structures. In inkjet printing, the recommended complex viscosity, for standard industrial printheads, ranges from 2–20 mPas [2,22]. However, *G*′ is more relevant for droplet and defect production due to the dominant role of elasticity in droplet formation [11]. The optimal range of properties is especially important within the printing frequency which typically lies between 2–7 kHz [16]. Nonetheless, the trend of evolution of *G*′ with respect to frequency is important, as the *G*′ curve is related to the molecular structure of the ink. In the literature, one can find the influence of the elastic ratio (*G*′/G*) and its relevance to ligament length and droplet production [11]. Although the established results in the literature serve immensely in understanding the basic inkjet-rheology relationship, the results obtained in the present work did not always correspond to already established quantitative descriptions. This might be primarily due to diversity in composition since different inks were used in this work. Therefore, this study focusses more on the qualitative analysis, to develop generalized relationships, especially in the case of inks with little or unknown composition information. To this end, extension of the Cox–Merz rule (a detailed explanation of which can be found in the literature [23]), and the assumption of a steady-state condition is applied.

Under the influence of surface tension, a jet of fluid finally disintegrates into droplets as per the natural tendency of fluids to attain a low-energy state [24]. However, in case of inkjet fluids, this phenomenon, evolving into Rayleigh–Plateau instability aids the droplet formation, by introducing an unstable form of equilibrium in the jets of fluid produced by capillary forces [25]. For droplet production in DoD printers, fluid (jet) columns need to be perturbed at deliberate points to terminate them at desired lengths and these perturbations can be induced by introducing peaks in the printing voltage waveform. The waveform designed for the PiXDRO (Süss Microtec SE, Garching, Germany) inkjet printer used in this work is shown in Figure 1. Since the actuators at the nozzle are piezo-electric in nature, the application of a pressure differential for drop production equates to the application of a voltage differential. Commercial printers supply this differential by applying a negative voltage followed by a positive voltage. However, in this work (due to hardware limitations) this differential is supplied by initially ramping the baseline voltage to 10 V and then lowering the voltage to 0 V, which would be the equivalent of a negative voltage in commercial printers.

The walls of the nozzle are thus mechanically flexed which results in the production of sinusoidal acoustic waves, the wavelength of which when matched with the circumference of the cylindrical fluid jet, generates ideal droplets. If their wavelength exceeds the circumference, the column of the fluid becomes unstable.

The success of producing droplets without defects, using the above-mentioned waveform will depend on the concentration of the polymer inks and the operating temperature. As literature has established, viscosity increases with concentration and rises exponentially after critical point, as described by Huggin’s and Martin’s equation [27]. Similarly with molecular weight (as according to the Mark–Houwink relation), viscosity shows a sharp increase after critical point, due to increased inter- and intra-bond interactions leading to entanglements in polymer chains [27]. With increasing temperature, the viscosity of a polymer solution decreases in general, with the shape of the slope of a flow curve being specific to a polymer type [28]. The rheological behaviour also changes due to aging factors. Certain changes are induced in the polymer with time, either due to foreign substances which react with it and change it permanently leading to ‘chemical aging’ or due to natural aging of the polymer which induces temporary changes (usually), referred to as ‘physical aging’. Structural changes due to aging have a detrimental impact on the behaviour of the polymer, especially its mechanical behaviour [29]. These changes can be brought in by photo- or thermal-oxidative reactions or due to contamination by metallic elements (needle tips) or hydrolysis, etc. [30]. Further explanations for the dependence of rheology on various parameters can be found in the literature [28,31].

**Figure 2:**
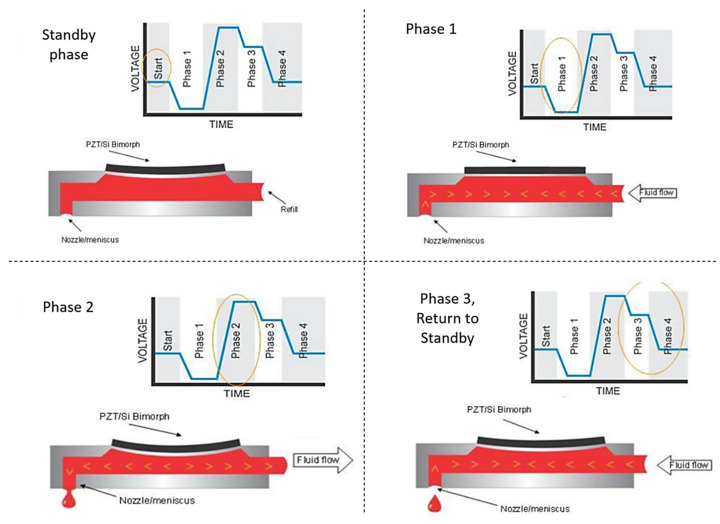
Different phases of the waveform experienced at the cartridge printhead [32].

Figure 2 shows different stages at the printhead and nozzle of a commercial printer and how each part of the waveform affects the way ink exits the nozzle. This waveform is slightly different from the one used in this work, shown in Figure 1, mainly due to the negative voltage applied in Phase 1, Figure 2, while in Figure 1, the voltage is ramped up and then brought down to compensate for the negative voltage, as mentioned earlier.

## 3. Experimentation—Methods and Materials

**Figure 3:**
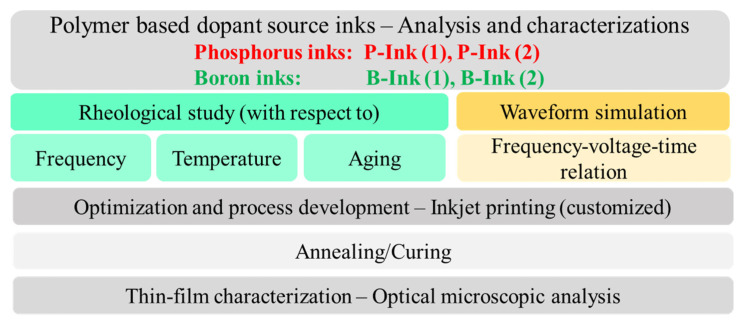
Experimental steps conducted in this work.

Figure 3 tabulates the experimental procedure followed in this work. Inks (obtained from commercial manufacturers) were characterized using a TriPAV rheometer (TriJet Ltd., Cambridge, UK) and a Picoscope (Pico Technology, Cambridgeshire, UK). The TriPAV rheometer allows analysis of the rheological behaviour of inks, in terms of their viscosity and elasticity, at high frequencies (up to 10 kHz), which is not possible with conventional rotational rheometers (which provide data in the 10–50 Hz [13] range only). In this work, four inks were analysed, two phosphorus- and two boron-based. Among the two P-inks used, difference lies in their polymer and dopant concentration, polymer chain lengths, concentration of organic additives, solvents, etc. Similar differences lie between the two boron inks. P and B dopant source inks have been categorized into ‘concentrated/dilute’ based on conventional arguments of base polymer concentration being ≥5% [33], and the polymer concentration in all used inks being above 20% at least, as indicated in Table 1 [34,35,36,37]. P- and B-inks (1) and P- and B-inks (2) were commercially purchased from Desert Silicon and Filmtronics, respectively.

Printing was conducted under an inert atmosphere inside a glovebox (GS GLOVEBOX System technik GmbH, Malsch, Germany) with pressure set to 0.8 mbar, and O_2_ and H_2_O levels to 3 and 0.7 ppm. While printing, the waveform as shown in Figure 1 was used. Although printing of dopant source inks can be conducted in an open atmosphere, a specific environment was maintained during the experimentation of this work, to achieve better control over various parameters such as humidity, temperature, pressure, etc., and to minimize the chances of contamination. Controlled atmosphere enabled sample preparation at constant and reproducible conditions and, hence, the extraction of significant correlations between the parameters of interest. The inks were stored as per the manufacturer’s recommendation.

### 3.1. Rheological Analysis

**Figure 4:**
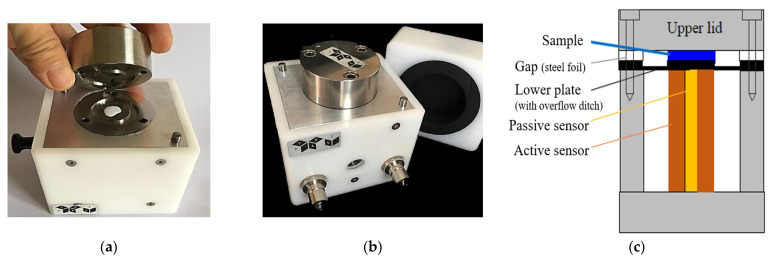
Schematics of the PAV rheometer: (**a**) sample (white coloured) placed on lower plate, (**b**) hermetically sealed plates, to be covered with the insulating top (present besides the device), (**c**) transverse section showing each part of the assembly inside the PAV rheometer [38].

As shown in Figure 4, one or two drops (approximately 0.1 mL) of ink were used to completely cover the lower plate of the PAV rheometer. The arrangement of the plates allowed for a hermetic seal between the plated set at a fixed gap (20–100 µm) with a steel shim. It consists of an active and a passive piezo sensor attached just below the bottom plate. 

The presence of air bubbles can be detrimental to exact and reliable measurements as air/dissolved gas significantly increases fluid compressibility, resulting in an increased pseudo elastic modulus (G′) of the viscoelastic fluid. This can significantly contribute towards drop compressibility in addition to dominating the elastic effects of the viscoelastic fluid [39]. In the printhead, the presence of air bubbles/dissolved gases can hinder the ejection of ink from the nozzles and therefore is undesirable in both situations. The device is provided with insulation around the top plate and the bottom plate is surrounded with a water jacket, the inlet and outlet of which can be seen in Figure 4b. This enabled us to measurements at temperatures other than at room temperature, using an external circulating thermobath heating system with a resolution for temperature sensing of 0.01 °C. The device performs well in a temperature range of 5 °C to 80 °C and produces precise results for viscosities ≥ 0.5 mPas. It allows the investigation of the jet-ability of inks in the frequency range of *f* = 1 Hz–10 kHz, covering the operating conditions of the inkjet printer. More details on the measurement principles can be found in the literature [10].

The analysis from the complex rheological flow curves can be used to design an inkjet printing procedure by selecting a favourable temperature, a time-dependent voltage waveform and a frequency range, all of which together, can help in developing the conditions required for inducing ideal ink behaviour. This ensures minimum nozzle clogging, the absence of satellites, tails, ligaments and threads and a homogeneous and precise deposition of ink onto the Si substrate.

### 3.2. Waveform Simulation

The inkjet printer used in this work was the PiXDRO LP50, equipped with a 10 pL Fujifilm Dimatix disposable cartridge. This inkjet printer provides for substrate alignment as well as print inspection and vision systems for real-time drop analysis. One of the key features of the PiXDRO LP50 inkjet printer, which makes it suitable for research and the development of inkjet processes, is the tunability of voltage and time of the waveform, which in turn can determine the shear-strain level applied to the ink during printing.

Simulating the voltage waveform using an oscilloscope (Picoscope by pico^®^ Technologies) [40] was especially helpful in relating the rheological behaviour to the printing/jet performance prior to actual printing. The equipment used is the same as that used for rheometry, except that an oscilloscope replaces the lock-in amplifier for waveform simulation. A continuous voltage (square wave signal) is applied to the lower plate of the device. Such input replicates the printhead channel/nozzle during waveform actuation. The output generated from the Picoscope is a measure of the damping ability of the ink, which in turn is related to the firing frequency and voltage applied at the printhead. In general, inks with high-dampening efficiency are printable at higher frequencies. Due to strong dampening effects, the fluid within the reservoir stabilizes within a short period of time. As a result, the next piezo-electric impulse can be applied sooner and the stimulus faces minimum obstructions to produce new stable droplets, making it possible to print at higher frequencies.

## 4. Results and Discussion

### 4.1. Rheometry

#### 4.1.1. Analysis with Respect to Frequency Sweep

**Figure 5:**
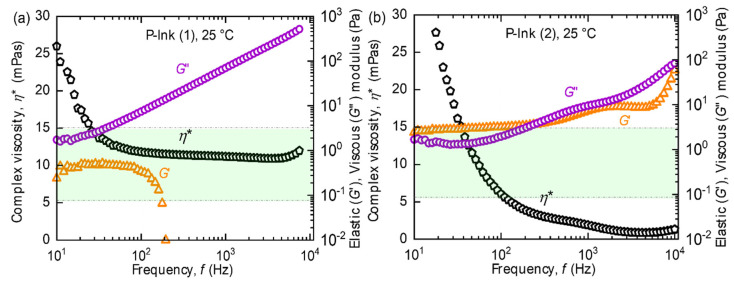
Flow curves show the complex viscosity (η*), elastic (*G*′) and viscous (*G*″) modulus measurements of (**a**) P-ink (1), and (**b**) P-ink (2) with respect to frequency (*f*) measured with the PAV rheometer [16]. Rectangular boxes in light green shade represent the range of rheological properties favourable for inkjet printing.

The flow curves of the P-inks, at room temperature, give an insight into the nature of the ink, varying with the frequency sweep, as shown in Figure 5. As per the manufacturer’s recommended inkjet printhead viscosity [22] range (highlighted in green in Figure 5) for P-ink (1) was observed in the entire frequency range, as in Figure 5a, but for P-ink (2) desirable values were limited in the frequency range *f_min_ ≥* 200 Hz to *f_max_ ≤* 1 kHz (Figure 5b). The favourable range of properties is specific to the printhead type thus allowing only limited fluids to flow smoothly. P-ink (1) exhibited near ideal behaviour, both qualitatively while printing and quantitatively in terms of the magnitude of η* as well as *G*′ and *G*″.

**Figure 6:**
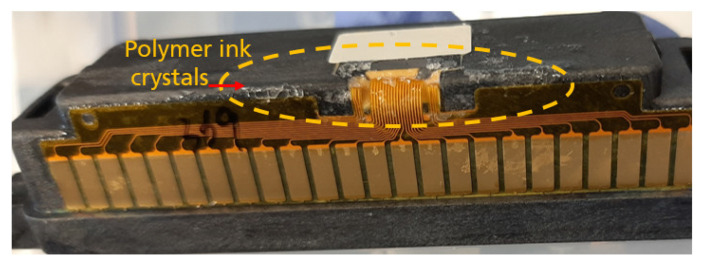
Strain-induced crystal formation for P-ink (2) around the printhead nozzle.

**Figure 7:**
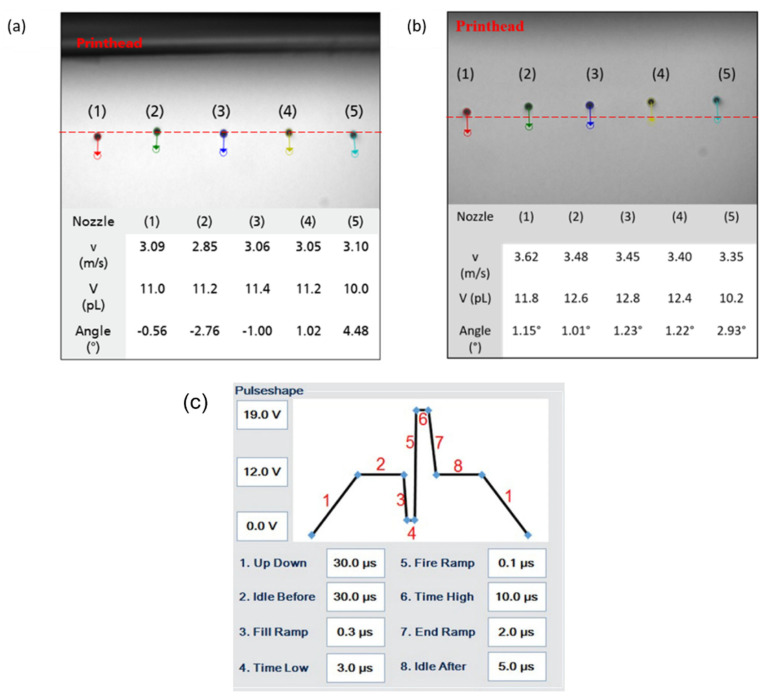
Dynamic analysis of (**a**) P-ink (1) and (**b**) P-ink (2) droplets captured by drop-watcher of the PiXDRO LP50 printer. The volume *V*, velocity *v*, and angle of the flying droplets quantify the printing behaviour. As shown here, controlling these parameters enables achievement of the precise printing of droplets onto the substrate. (**c**) The waveform used to obtain the droplets in (**a**,**b**).

P-ink (1), which is mostly Newtonian, showed weaker elastic effects, compared to other inks. From the trend of *G*′, we can infer that the structure of the polymer remains unchanged up to a threshold, after which it starts deforming, probably due to enhanced straightening of bonds due to strain and, hence reducing mechanical interactions. The viscous component, *G*″, which is always higher in magnitude than the elastic component, *G*′ represents the dominant fluid nature of the ink. Inks with such features have been mostly found to behave favourably during printing. P-ink (2) (Figure 5b) exhibited completely different behaviour compared to P-ink (1). η* was less than 5 mPas, i.e., on the lower side of the recommended range, (i.e., 2–20 mPas). The curve for *G*′ implies a rigid polymer structure, inheriting its rigidity from strain hardening caused by strain-induced crystallization or entanglements caused by long silicate [37] backbone chains [41]. This is in good agreement with the Cox–Merx rule, a detailed explanation of which can be found in the literature [42]. Similar results of different polymer solutions (mono- and bi-disperse), abiding by the Cox–Merx rule has been explained by Wen et al. [43]. Moreover, the cross-over between *G*′ and *G*″ implies that the ink was not predominantly fluidic in nature, such as observed with P-ink (1). Indeed, strain-induced crystals were found around the nozzle, as shown in Figure 6, which serve as evidence. The viscoelastic response of a polymer is best understood using the Voigt’s and Maxwell’s elements of Burger’s model of equivalent mechanical circuit [44]. As explained by J.D. Ferry [45], usually, interactions and entanglements amongst the polymer chains are secondary in nature. As the frequency increases, which is equivalent to saying that the shear-strain rate increases (following the Cox–Merz rule), the stiffness of the polymer increases before reaching a maximum, like a Hookean elastic material. However, like P-ink (1), the printing performance of P-ink (2) was stable with almost ideal droplet formation, as can be seen in Figure 7b, when printed in the highlighted frequency range (in the rectangular box, Figure 5b). As is evident from Figure 7, all the nozzles in both the inks were active, which is the ideal working situation. The droplet features explain the printing features, such as, droplet volume which determines the droplet spread on the substrate and the precision of the desired design. A droplet volume of 12 pL is ideal considering the nozzle size of a 10 pL printhead and the minimum resolution of the printer.

The nozzle experiences a complex combination of the frequency of the waveform and the firing frequency, generating the required shear strain to deform the fluid column repeatedly at a particular frequency, which is referred to as the ‘firing frequency’. It is directly dependent on the time required to completely break up the fluid ligaments into each droplet after their jetting. As is evident in Figure 5, the P-ink (1) was less elastic compared to P-ink (2), and therefore could be printed at higher frequencies due to less waiting time. It was observed that with a resolution of 600 dpi, the desired droplets were produced at a printing speed of 300 mm/s which is equal to a firing frequency *f*~7 kHz. Whereas in the case of P-ink (2) at 600 dpi, the ideal printing speed was 100 mm/s, equating to a firing frequency *f* = 2.3 kHz. The timing of the pressure waveform, shown in Figure 7c, was designed in accordance with the frequency range exhibiting the desired elasticity values (Figure 5), according to the rheology of the inks. Figure 5a shows that the frequency range of 1–6 kHz provided the desired elasticity values for P-ink (1). Scaling the timescale from the rheometer to the printhead, the timescale of the pressure waveform was kept between 8–80 µs. Similarly, for P-ink (2), illustrated in Figure 5b, the desirable frequency range was between 200 Hz–1 kHz and equates to a timescale of 50–250 µs. The voltages of the pressure waveform, as mentioned in Section 2, are dependent on the complex viscosity. Inks with a viscosity in the desired regime between 5–15 mPas, such as P-ink (1), are easily printable at approximately 20 V, for ideal droplets with no associated tails, ligaments or satellites. This is also close to the default actuation voltage of the LP50 printer—25 V, and the droplet size is close to the nominal volume of the printhead (10 pL). Section 4.2.1 presents a more detailed explanation in the last paragraph.

The flying velocity determines the homogeneity of the print. Dissimilar flying velocities can lead to some areas being left unprinted, with excessive ink being deposited elsewhere. The angle of the flying droplets plays a significant role in determining the precision of the design. Too large an angle would imply a greater deviation from the ideal orthogonal direction to the substrate and hence would lead to imperfections in the printed structures. This can be a serious drawback especially for obtaining a small feature size, as previously published [16].

**Figure 8:**
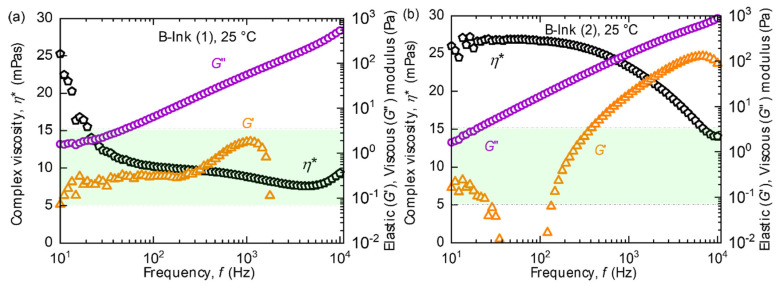
The complex viscosity (η*), elastic (*G*′) and viscous (*G*″) modulus measurement of (**a**) B-ink (1), and (**b**) B-ink (2) with respect to frequency (f) utilizing the PAV rheometer. Rectangular boxes in light green shade represent the range of rheological properties as recommended by supplier, for inkjet printing.

The printing behaviour of the B-inks was contrary to that of the P-inks. Comparing the rheological characteristics of B-ink (1)-Figure 8a, with P-ink (1)-Figure 5a, at 25 °C they appear very similar. η* shows a small negative slope; however, within the inkjet printing frequency range, the values are within the supplier recommended region.

**Figure 9:**
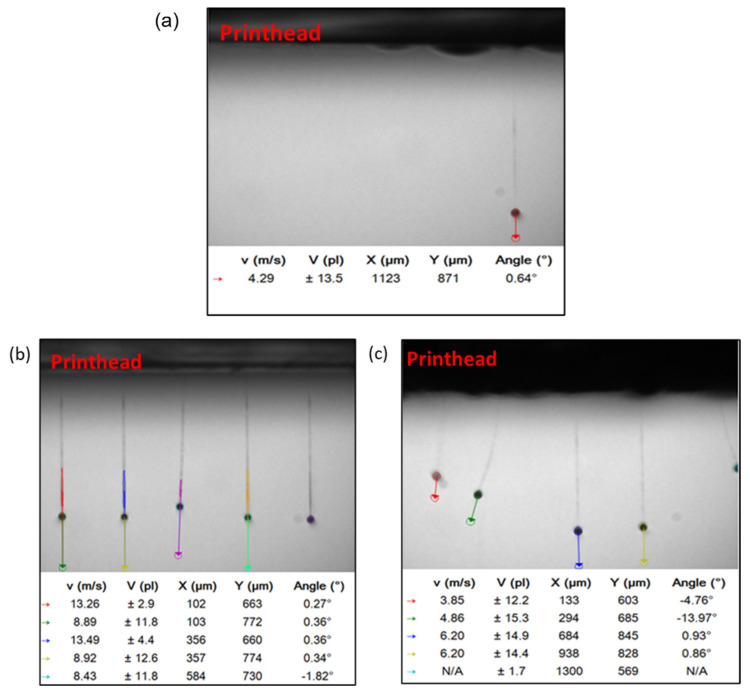
(**a**) Dynamic analysis of B-ink (1) droplets by drop-watcher of the PiXDRO LP50 printer showing clogged nozzles produced by using the waveform shown in Figure 7 (**c**). (**b**) Long tails of large droplets produced due to an increased printhead temperature of 40 °C and (**c**) diverging droplets with random flight directions producing unwanted printed structures using an increased voltage of 60 V.

Despite the similar rheological behaviour, B-ink (1) was unsuitable for printing unlike P-ink (1), and their jetting results were found to be quite the opposite (Figure 9a). Most of the nozzles with B-ink (1) were found to clog, with no droplet ejection. Therefore, rheological analysis of the inks at the jetting temperature (25 °C) alone may not be sufficient to predict the jetting behaviour.

In some cases, the droplets can coagulate around nozzles and fail to detach allowing only a few nozzles work. However, printing can still be conducted by selecting the only working nozzles manually, while setting up the system, which is not an ideal situation as it takes longer and can be avoided by adjusting the printhead temperature, increasing the piezo voltage or changing the waveform. Such changes, however, may lead to an ejection of large volumes of ink leading to the formation of defects, such as satellites and long tails, as can be seen in Figure 9b,c. The presence of such defects is a setback to the precision promised by inkjet technology. Therefore, to understand the ink behaviour further, an investigation based on different parameters (temperature and aging) was conducted, as discussed in later sections.

**Figure 10:**
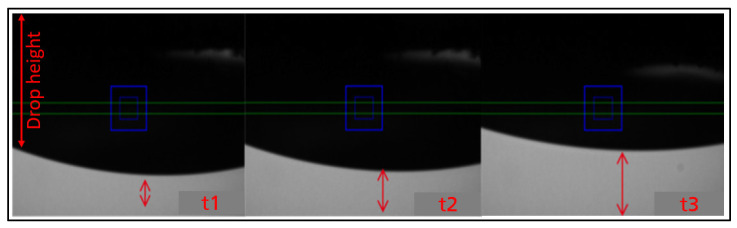
Coalesced drop of B-ink (2) retracts back into the nozzle, even after being subjected to 60 V and 5 s purging, failing droplet formation.

**Figure 11:**
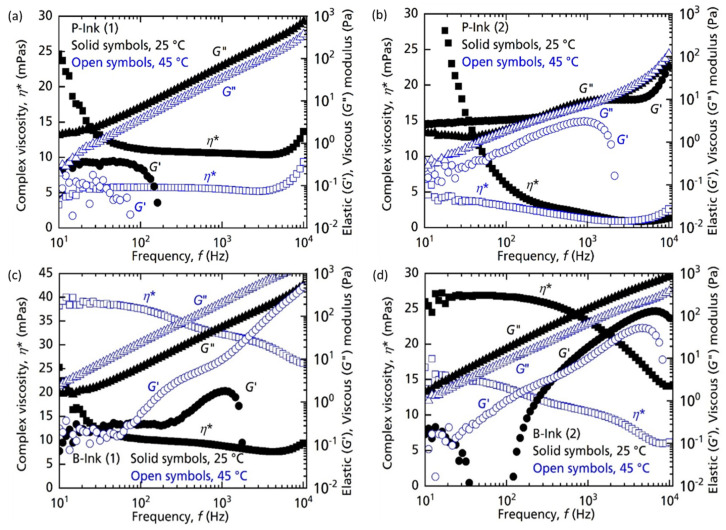
Evolution of the rheological flow curves with a rise in temperature. Complex viscosity (η*), elastic (*G*′) and viscous (*G*″) modulus measurements of (**a**) P-ink (1), (**b**) P-ink (2), (**c**) B-ink (1) and (**d**) B-ink (2), with respect to frequency (*f*) at 25 °C and 45 °C as measured by the PAV rheometer.

**Figure 12:**
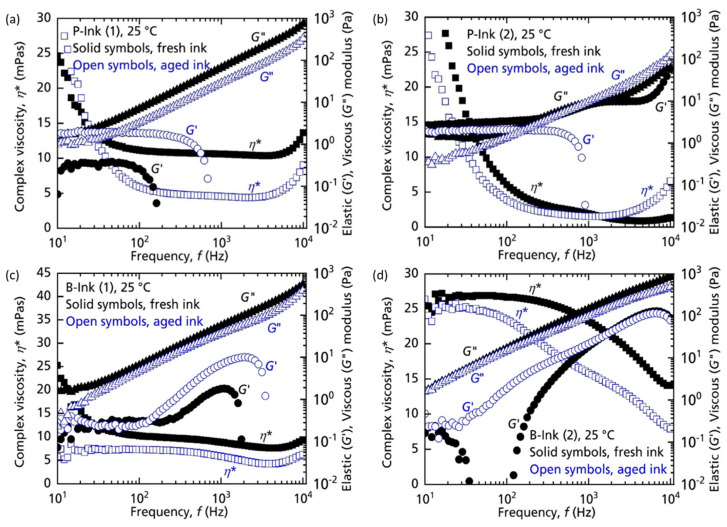
Rheological flow curves exhibiting aging effects. Complex viscosity (η*), elastic (*G*′) and viscous (*G*″) modulus measurements of (**a**) P-ink (1), (**b**) P-ink (2), (**c**) B-ink (1) and (**d**) B-ink (2) with respect to frequency (*f*) at 25 °C utilizing the PAV rheometer.

The parameters lie outside the desired range by a large factor, causing difficulties in droplet production. The complex viscosity is approx. η* ≈ 25 mPas, which is five units higher than the upper limit. If the complex viscosity was the only deciding factor, adjusting the printhead temperature or increasing the applied voltage (which increases the strain rate) alone would reduce the viscosity. However, as known already, the elastic component of the storage modulus *G*′ plays a stronger role in determining the printability, which cannot be changed sufficiently in this case through external parameters alone, given the large margin from the ideal range. Here, *G*′ can only be changed effectively by changing the chemistry of the polymer, i.e., reformulating the inks. This proved to be a limitation in tuning the rheology in this work. The set of data points for the first few tens of Hertz (frequency) can be neglected since scatter can arise due to instrument errors. As a result of intense interactions and its strong elastic nature, B-ink (2) failed to produce droplets. Intuitively, a high voltage (up to 100 V) and a high purging time (up to 5 s) were employed to force droplet formation. The strong pressure yielded a big drop resulting in nozzle plate flooding, which slowly retracted its way back into the cartridge as shown in Figure 10. All the inks showed a conventional shape of η* curve captured at different stages. For P-ink (1), P-ink (2) and B-ink (1) η* had already left the ‘zero-shear rate viscosity’ [46] (*η_0_*) and showed a linear downward slope towards a constant high-shear rate viscosity (*η_∞_*). At both *η_0_* and *η_∞_*, polymer molecules are fully aligned and produce the least resistance to flow. However, in the middle region of transition entanglements are expected to be present in large amounts, as explained in the literature. As can be observed in all the flow curves for B-ink (2) (Figure 8, Figure 11d and Figure 12d), η* stayed in the upper plateau for longer frequencies. In the printing frequency range of 2–7 kHz the ink exhibits a transition phase, and thus due to presence of many entanglements the resistance to flow was the greatest. The higher polymer concentration and possible interaction between the polymeric ingredients could also cause such behaviour, besides high molecular weight.

#### 4.1.2. Analysis with Respect to Temperature

Polymers exhibit Arrhenius or Williams–Landel–Ferry (WLF) dependence of complex viscosity on the subjected temperature and its relevance to the glass transition temperature (*T_g_*) [31]. However, in general, the rheological properties reduce in magnitude upon increasing the temperature, as is also experienced with dopant source inks used here. As the temperature increases, the thermal energy can break longer polymer chains into smaller ones as well as disentangling them. This will generate a corresponding response from the ink at lower shear-strain rates (frequency). As shown in Figure 11, P-ink (1) exhibited such a direct relation for all the three properties upon increasing temperature. η*, *G*′ and *G*″ decreased by a noticeable amount as the temperature increased from 25 °C to 45 °C. η* reduced to ~5 mPa, still within the printable range. The complex viscosity in fact showed a stronger Newtonian behaviour up to approximately 7 kHz. Values towards the extreme ends were neglected due to inertia effects in the rheometer. The elastic component of the storage modulus *G*′ showed a scatter of data at 45 °C, nearly following the same trend as at 25 °C. Similar to *G*′, *G*″ also reduced in magnitude only, keeping the trend of evolution the same. Such simple changes in the properties exhibited a conventional polymer nature of the dopant source inks. Further detailed discussion can be found in the literature [29,31].

Like P-ink (1), P-ink (2) also showed simple changes of the parameters upon increasing temperature. Due to such behaviour, we could produce near ideal droplets, free from ligaments and satellites for P-ink (1), with a 25 V printhead voltage, at 25 °C, whereas for P-ink (2), near ideal droplets were produced at a higher voltage (35 V). A higher voltage is equivalent to increasing the pressure, both translating to higher energy. At 45 °C, we observed a categoric separation of the *G*′ and *G*″ curves of P-ink (2), which as observed in Figure 5b have a cross-over at 25 °C, implying a well-behaved response of ink under a strong impulse (equivalent to temperature of 45 °C, at least). Moreover, for inks containing a substantial amount of solvent (>20% [36]), a high voltage is preferred over a high printhead temperature to avoid evaporation of the solvent which can have a negative impact of performance by increasing the ink’s viscosity. Contrary to the conventional nature of P-inks, the rheological properties of B-ink (1) increased in magnitude with increasing temperature. As shown in Figure 11c, at 25 °C, B-ink (1) was not radically different from P-ink (1) but, at 45 °C, the magnitude immensely increased. η* increased from an approximately constant value of 6 mPas to a range, varying from η*_min_ = 30 to η*_max_ = 40 mPas, changing from near Newtonian at 25 °C to non-Newtonian at 45 °C. A continuously increasing trend of *G*′ at 45 °C suggests a clear increase in elastic strength. The presence of small quantities of polymer has been found to be helpful in droplet production [47], but in this case, a large amount of polymer sabotages the fluidic nature. High viscosity values can be countered using high temperatures or voltages, which, however, cannot change the elasticity substantially. Due to this limitation, increasing the toughness (increasing *G*′) led to a failure in printing. Such unexpected behaviour of the polymer can imply several underlying factors. One possible reason could be cross-linking of polymers due to the higher thermal energy available at higher temperatures, as is a common trait of polymers [31]. Cross-linking can provide greater rigidity to a polymer, increasing its resistance to deformation. Moreover, at higher temperatures, inks can witness molecular aggregation and polymerization itself can continue further, not necessarily leading to cross-linking. Both situations can have a similar impact on the rheological properties of the inks, increasing their magnitude. B-ink (2), on the other hand, showed conventional polymer behaviour. As shown in Figure 11d, although the properties reduced, they were still too high considering the workable range of the inkjet printer.

#### 4.1.3. Analysis with Respect to Aging

Inks were stored as per manufacturer’s instructions—isolated in a dry environment at room temperature for two months and rheological characterization was repeated. Given the care taken while storing the inks, the rheological changes suggest a likely occurrence of ‘physical aging’. The kinetics, mechanism, and impact of physical aging on several properties has been explained in great detail in the literature [29]. The changes developed in inks after physical aging are expected to be polymer specific. The response of ink depends as much on aging time and temperature, as it depends on its polymer concentration, chemical potential of the molecules and entanglement density. There are reports of concentrated polymer solutions showing exponential degradation leading to a reduction in their viscosities [48] while at the same time their dilute counterparts exhibit no degradation. It has also been reported that a mere enhancement in dissolution of the additive/solvents leading to the disentanglement of polymer blobs into simple polymer chains can also result in reduced viscosities [49]. While considering the changed dynamics of the polymer inks due to aging, the application of strain–stress on aged inks leads to different outcomes compared to fresh inks. Given the high-stress conditions at the nozzles there is a possibility of change of the linear viscoelastic behaviour to non-linear behaviour during printing. The inextricable connection between physical aging and non-linearity can produce unforeseen responses and is a complex problem to resolve [29]. In many polymers such changes can bring about ‘mechanical enhancement’, which leads to an increase in the moduli of polymers. Such results have been reported [50], although for glassy polymers, but the relations between the viscoelastic parameters can be extrapolated from polymer melts to polymer solutions, as has been explained in the literature [51]. In the case of P-ink (1) and B-ink (1) we saw similar outcomes, where stress application during testing might have led to mechanical enhancements thus increasing *G*′ values, as is evident in Figure 12a,c. P-ink (1) showed a reduction in complex viscosity after aging for two months. The curve retains Newtonian shape for most part. It must be noted that this ink was near its expiration, which could have further induced chemical changes. Contrary to P-ink (1), P-ink (2) did not exhibit drastic changes in its properties upon aging, implying chemical stability. As in Figure 12c, in B-ink (1) η* reduced in magnitude while maintaining the shape of the curve. The elastic component *G*′ increased in magnitude while the viscous component *G*″ only slightly reduced. As for P-ink (1), B-ink (1) was also near its expiration date, implying possible chemical changes, which can change the elastic modulus. Nonetheless, a factor as simple as evaporation of low-boiling-point solvents (such as ethanol, refer to Table 1) could also be an eligible explanation. B-ink (2), Figure 12d, showed a reduction in η*, as well as changes in the nature of the ink, from Newtonian-like to non-Newtonian. The stability of *G*′ and *G*″ implies possible changes in polymer morphology leading to a lower complex viscosity, probably due to the disentanglement of chains.

All the rheological characterization was conducted while printing dopant layers on silicon wafers during the experiment.

### 4.2. Inkjet Printing

#### 4.2.1. Waveform Simulation

**Figure 13:**
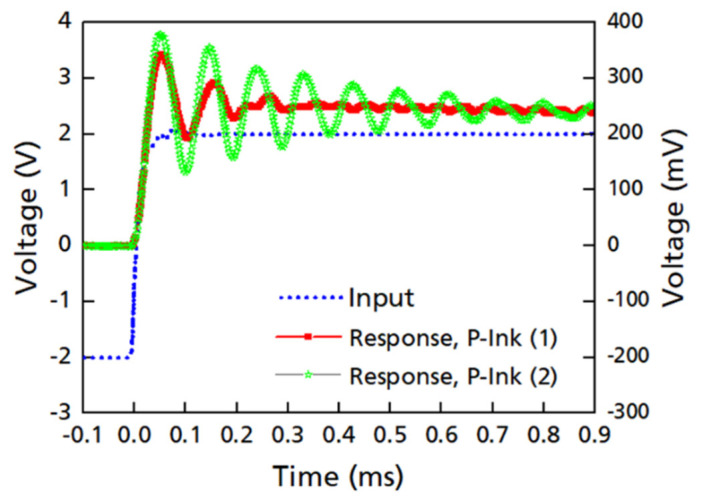
Waveform simulation (voltage vs. time) obtained from the Picoscope at 25 °C for P-ink (1), and P-ink (2).

The presented waveform simulation demonstrates a crucial link between rheology and printing by replicating the impulse generated by the time-dependent voltage waveform at the piezo-electric nozzle head (in inkjet) in the form of rectangular voltage signals using a Picoscope. Figure 13 captures the response of the inks in the form of sinusoidal waves (red and green), while the blue wave is the input.

Red profile: rapid meniscus damping, and only needs a shorter waveform wait time. Green: longer transient time required for oscillation to dampen; thus, a longer waveform wait time. To relate the sinusoidal output of the inks to their rheology, it is imperative to compare them to the default waveform of the PIXDRO LP50 inkjet printer, as shown in Figure 1, which lasts for 0.1 ms (approx.). The total waveform time 0.1 ms is smaller than the time at which the signal in the wave subsides, i.e., 0.3 ms for P-ink (1). This means there are residual/secondary vibrations in the ink (cartridge reservoir) after the waveform ends, which suggests the presence of meniscus oscillations post-jetting. If the next waveform signal is applied before the residual waves subside, the new signal hits the ink before the effects of the previous signal end. As a result, the length of the jet des not resonate with the Rayleigh length and thus the droplets produced contain defects in the form of tails, ligaments or satellites. For P-ink (1) the waveform was therefore redesigned and stretched out to slightly more than 0.3 ms by reducing the printing frequency. As shown in Figure 13, for P-ink (2), the wave took much longer to subside, approximately 1 ms. This is in strong agreement with the higher *G*′ magnitude as observed for P-ink (2), compared to P-ink (1) and explains why a higher voltage was required to produce droplets. The higher voltage ensures that the ink was constantly under higher pressure helping overcome the stronger elasticity of the ink. Moreover, the printing frequency was accordingly adjusted to make the waveform last longer. Both effects combined ensure that the droplets produced are near to ideal droplets, as shown in Figure 7b.

The simulation responses from P-inks, in general, appropriately reflects their rheological behaviour. The less time for dampening for P-ink (1) reflects the comparatively stabler nature of the ink than P-ink (2). This goes in parallel with the flow curves, Figure 5, Figure 11 and Figure 12, which suggest the Newtonian nature of P-ink (1), less elastic effects and predominant fluidic nature, while for P-ink (2), a stronger elastic modulus and an equivalent viscous modulus strength (not allowing a fluidic nature to dominate). However, it must be noted that even though simulation provides vital information regarding ink behaviour and the designed waveform, it does not take into consideration some important factors which are experienced while printing, such as undesirable heat production, solvent loss, nearness of critical points (critical concentration, molecular weight, phase transition, etc.), strain hardening and strain-induced crystallization, etc., limiting the entire imitation of the operating conditions. Therefore, simulations at best represent the behaviour of inks with the assumption of laws being followed conventionally, without any peculiarities.

As a result, pertaining to the peculiar flow curves of B-ink (1) as is evident from Figure 8, Figure 11 and Figure 12, simulation outcomes did not represent the actual situation. For B-ink (2), although the parameters were very stable throughout and rather insensitive to the changes in frequency, temperature and aging, the magnitude of the parameters being too far from the ideal range, renders the ink unusable. Results for B-ink (1) and B-ink (2) were also obtained. However, due to the criticality of the parameters for these two inks, simulation at 25 °C did not replicate the entire material dynamics and thus was not as helpful for the P-inks.

While optimizing the waveform, to reach the jet length required for Rayleigh instability, the position of the peak needs to be shifted to the left or right in the trapezoidal waveform shown in Figure 1, depending on the damping ability of the ink. The ability of inks to damp these vibrations varies according to their polymer—molecular weight, concentration, etc. To ensure ideal drop production the next pulse should be applied only after enough time elapses to ensure substantial damping. The time it takes to subside the vibrations is the ‘waiting time’. The duration of the waiting time can be calculated through waveform simulations [16]. Shifting the ramp position in the waveform shifts the pinch-off time (Figure 2), which eventually determines the length of the jet termination. While shifting the peak, the overall timescale of the waveform must be taken into consideration. This is related to the frequency of the rheometer scaled by a factor of 20, since, while the active length for the PAV rheometer (TriPAV, Trijet Limited, Cambridge, UK) is 20 mm (diameter of plate), for printheads it is usually approximately 1 or 2 mm (area of the cross-section which experiences mechanical perturbations, including the nozzle area). The timescale thus chosen represents the range of rheological properties associated with the frequency range.

#### 4.2.2. Optical Characterization

**Figure 14:**
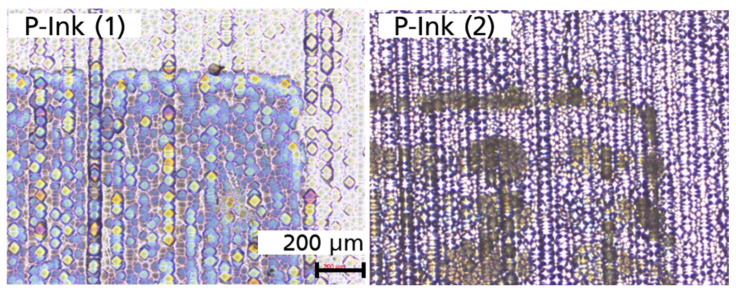
Optical microscope images captured at a resolution of 200 µm, after high-temperature annealing in a tube furnace, for P-ink (1) and P-ink (2), printed on a saw-damage-etched surface of the CZ-Si wafer at a resolution of 600 × 600 dpi [16].

Printed squares were successfully obtained from P-ink (1) and P-ink (2), as exhibited by the well-defined edges in Figure 14. The Si substrate of the CZ-Si wafer had a saw-damage-etched surface. Conducting printing at resolution of 600 dpi, the squares produced were sharp-edged (for a large area) and well defined.

The following parameters were used for the successful printing of P-ink (1):

- print resolution—from 300 dpi to 600 dpi;
- firing frequency—from *f_min_* = 2 kHz to *f_max_* = 7 kHz;
- printing speed—100 mm/s to 300 mm/s.

**Figure 15:**
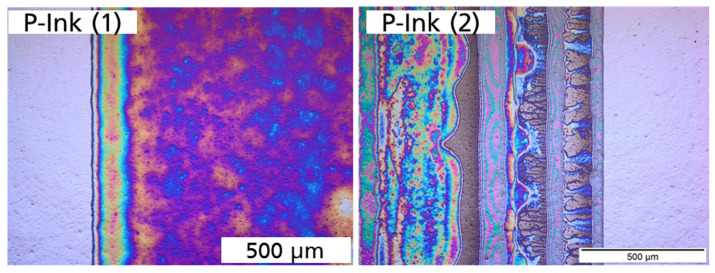
Optical microscope images captured at a resolution of 500 µm, after high-temperature annealing in a tube furnace, for P-ink (1) and P-ink (2), printed on a shiny-etched surface of the FZ-Si wafer at a printing resolution of 600 × 600 dpi [16].

From the micrographs captured at 200 µm and 500 µm resolution, the precision of the shapes is evident as seen in Figure 14 and Figure 15.

There were inhomogeneities in the form of different colour shades for P-ink (1) and an accumulation for P-ink (2) which could have arisen due to a differing rate of evaporation across the surface and substrate–ink incompatibility interactions, respectively. There are various routes through which this issue could be addressed, such as plasma treatment of the Si substrate surface, passivation, etc.

## 5. Summary and Conclusions

The work covered in this paper studied the rheological behaviour of two phosphorus and two boron dopant source polymer-based inks. Changes in their complex viscosity (η*)*,* elastic (*G)* and viscous *(G*″) components of the shear modulus (G*), upon changing the frequency (or strain, as per the Cox–Merz rules extension), temperature and the age of the inks were studied. Inks which showed complex viscosity ranging from η*_min_ ≥ 2 mPas to η*_max_ ≤ 20 mPas, an elastic component of the shear modulus, *G*′ ≤ 20 Pa and a linear trend for the viscous component of the shear modulus, *G*″ (always higher than *G*′) in the frequency range, i.e., *f*_min_ ≥ 2 kHz to *f*_max_ ≤ 7 kHz, at room temperature, were found to produce well-behaved droplets with minimal defects.

Well-behaved inks were found to be stable at all temperatures, such as P-ink (1) and (2), showing a non-increasing *G*′ curve and a η* ≤ 15 mPas at RT, and both the properties lower with increasing the temperature to 45 °C and upon aging, as per conventional laws. Inks which did not obey conventional polymer laws presented complications during printing in terms of stability of the jets of inks, production of droplets from only a few nozzles due to coagulation and entanglement of the polymer inks, different volumes of droplets from different nozzles, non-orthogonal direction of fall of droplets onto the substrate, presence of tails and satellites following the main droplets and, in some cases, complete failure of droplet production. These inks, such as B-ink (1), behaved contrary to conventional polymer nature with increasing η* with temperature and aging.

Rheological behaviour, with respect to temperature and aging, reflect the structural changes within a polymer. Changes distinct from the norm suggest the possibility of a nearness of critical parameters of the polymer and thus an undesirable shift in its properties. Therefore, inks which are chemically stable (with temperature and age) are more reliable considering the application of an optimized waveform and the successful reproducibility of printing. Further confirmation of this hypothesis was confirmed by voltage waveform simulation, which aided in the understanding of the voltage and time scale requirements depending on the dampening ability of the ink and further designing a waveform according to the ink and successfully producing droplets with near ideal features. In a perfect case, one would prefer an ‘ideal’ viscoelastic ink that gives good peak amplitude and shorter peak time and faster sinusoidal damping (shorter transient relaxation time). Such ink requires a lower drive voltage, a shorter pulse width and wait time in its waveform to eject the drop. This would be an ideal fluid for reliable jetting at higher frequencies.

Observations made on diverse parameters, such as in this work, imply the importance of polymer structures (molecular weight/concentration/etc.) and their role in determining the rheological behaviour of such inks. A further step in making inkjet printing a success would be efficient engineering of polymer structures such that ink abnormalities become avoidable, while at the same time the versatility of inkjet printing is fully exploited.

## Figures and Tables

**Table 1:** Classification of polymer-based dopant source inks in alcoholic solvents.

Composition	Organometallic (Silicate Polymer) Solutions	Polymer in Alcohols	Organometallic (Silicate Polymer) Solutions	Polymer in Alcohols
**Ink type**	P-ink (1)	P-ink (2)	B-ink (1)	B-ink (2)
**% Polymer**	<20%	>20%	<20%	>20%
**Solvent**	Ethanol + Glycerol	Ethanol (Aqueous)	Ethanol + Glycerol	Ethanol (Aqueous)

## Data Availability

Not applicable.
